# Longitudinal Associations Between Loneliness and Self-Rated Health

**DOI:** 10.1093/geroni/igab046.2250

**Published:** 2021-12-17

**Authors:** Ketlyne Sol, Simon Brauer, Toni Antonucci

**Affiliations:** University of Michigan, Ann Arbor, Michigan, United States

## Abstract

Cross-lagged structural equation models examined the bidirectional association between loneliness and self rated health over three time points. We adjusted for age, gender, network size, and depressive symptoms at baseline. At baseline, the sample was 28% Black and 40% male. Average age at time 1 was 46 years, 56 years at time 2, and 63 years at time 3. Results indicated that loneliness at time 1 was associated with loneliness at time 2; loneliness at time 2 was associated with loneliness at time 3. We had similar findings for associations among self rated health. However, only one of the cross-lagged paths was significant. Specifically, more loneliness at time 2 was associated with worse self rated health at time 3. These associations did not vary across black and white race. Findings indicate that loneliness at later midlife may be detrimental to later life health, regardless of race.

